# Metabolomics Reveals Rubiadin Accumulation and the Effects of Methyl Jasmonate Elicitation in *Damnacanthus major* Calli

**DOI:** 10.3390/plants13020167

**Published:** 2024-01-08

**Authors:** Hyejin Hyeon, Eun Bi Jang, Sung Chun Kim, Seon-A Yoon, Boram Go, Jong-Du Lee, Ho Bong Hyun, Young-Min Ham

**Affiliations:** Biodiversity Research Institute, Jeju Technopark, Seogwipo, Jeju 63608, Republic of Korea; hhj2065@jejutp.or.kr (H.H.); jeb311@jejutp.or.kr (E.B.J.); sckim@jejutp.or.kr (S.C.K.); yoonsa33@jejutp.or.kr (S.-A.Y.); boram01@jejutp.or.kr (B.G.); jdlee1015@jejutp.or.kr (J.-D.L.); hyebong@jejutp.or.kr (H.B.H.)

**Keywords:** *Damnacanthus major*, callus, elicitation, methyl jasmonate, rubiadin, metabolomics, multivariate analysis

## Abstract

Callus suspension techniques have been considered attractive for improving bioactive metabolite productivity; methyl jasmonate (MeJA) is a widely used elicitor for stimulating synthetic pathways. In this study, a multivariate analysis-based metabolomics approach was employed to investigate the primary and specialized metabolites in the leaves, unelicited calli, and 100 or 200 μM MeJA elicited calli of *Damnacanthus major*. Rubiadin, a powerful anthraquinone with various therapeutic properties, was only identified in *D. major* calli, accumulating in a MeJA elicitation concentration-dependent manner. Callus cultures also contained high levels of amino acids, sugars, and phenolic compounds, indicating energy metabolism and metabolic adaptation responses for proliferation and stabilization. Regarding MeJA application, elicited calli contained higher amounts of quinic acid, kaempferol, and glucose with lower amounts of sucrose and raffinose than those in the unelicited control, which were closely related to protective mechanisms against MeJA. Moreover, excessive elicitation increased the asparagine, fructose, and raffinose levels and decreased the glucose and sucrose levels, which was ascribed to increased activation of the aminoacyl-tRNA biosynthesis pathway and wider utilization of glucose than of fructose after sucrose degradation. These results will be useful for optimizing plant cell culture techniques to achieve high production rates for valuable specialized metabolites.

## 1. Introduction

*Damnacanthus major*, an evergreen shrub of the Rubiaceae family, is approximately 30–70 cm tall, and grows naturally in warm regions, such as Korea (southern), China (southern), Japan (southern), Thailand, and India [1,2]. Recently, the genetics [3,4], medicinal material quality control [5], and synthesis of new metabolites and drug prototypes [6] have been studied in the Rubiaceae family owing to the diversity of chemical constituents. Among various secondary metabolites produced by the Rubiaceae family, anthraquinones are biologically active with anti-inflammatory, antioxidant, anticancer, hemostatic, and antibacterial activities [7]. Phenolic compounds are dominant in the Rubiaceae family and are medicinally important owing to their anticancer, anti-inflammatory, hepatoprotective, antibacterial, and antiviral properties [8]. However, increased demand for these bioactive plant-derived compounds may lead to excessive use of plant resources, ultimately causing biodiversity loss [9].

To overcome this problem, callus cultures can be used for the industrial production of rare or endangered plants, as well as those that are difficult to secure because of their small size or small number [10]. Callus cultures can produce specialized metabolites at a faster rate than that of traditional cultivation and are performed under controlled conditions without being affected by various environmental factors (seasonal changes, microbial diseases, pests, and geographical constraints); therefore, their functional substances are continuously produced with consistent quality [11]. Elicitation is an effective strategy for increasing the biosynthesis of bioactive compounds and enhancing biomass in plant cultures in vitro [12]. Methyl jasmonate (MeJA) is a powerful abiotic elicitor. Exogenous MeJA treatment of in vitro cultures increases bioactive compound biosynthesis, antioxidant enzyme activity, and defense-related gene expression [12,13]. Notably, Komaraiah et al. reported that anthraquinone production was significantly increased with MeJA elicitation than with polyunsaturated fatty acid elicitation [14].

Metabolomics, an omics approach for studying the metabolite changes in biological systems, has drawn significant interest in systems biology, as it can reflect the reactions of plant cells or tissues to various abiotic stresses [15]. Primary metabolites are compounds involved in essential physiological functions, including growth, development, and reproduction [16]. Unlike the primary metabolites commonly observed in all plant species through central metabolism, specialized metabolites are biosynthetic end products that play significant roles in defense by enhancing resistance against biotic and abiotic stresses [16]. Specialized metabolites are commercially utilized in the food and pharmacological industries because of their structural diversity and biological functions [17]. Therefore, metabolic profiling of both primary and specialized metabolites can provide a comprehensive understanding of the biological pathways occurring in plants under various stresses.

Most plant cell culture techniques focus on the presence of bioactive compounds; however, a combination of primary and specialized metabolite profiling is rare. Further, little information is available regarding the metabolic profiles of Rubiaceae family cell cultures, especially the calli of *D. major*, under stress conditions. Therefore, the present study aimed to identify the primary and specialized metabolite differences among *D. major* leaves, unelicited calli, and calli elicited with 100 or 200 μM MeJA. The findings of this study can be helpful for increasing biomass and specialized metabolite production in calluses in vitro compared with that in explants through elicitor treatment. Further, the metabolic profiling strategy adopted in the current study can provide a reference for future plant biotechnology approaches on the effects of MeJA elicitation and determine the potential relationship of primary and specialized metabolites with the plant stress response in *D. Major* calli.

## 2. Results and Discussion

### 2.1. Effects of Methyl Jasmonate on Callus Proliferation

**Figure 1:**
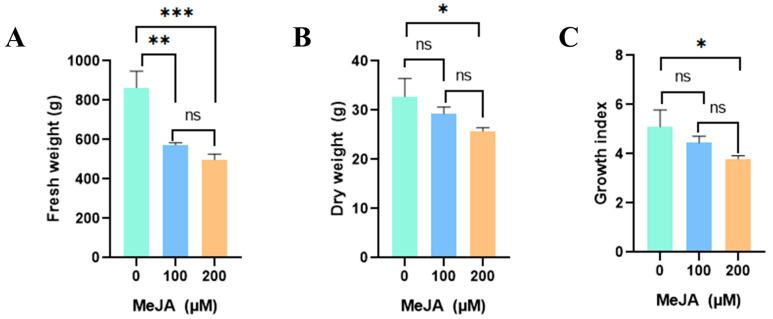
Biomass accumulation in response to different methyl jasmonate (MeJA) concentrations in the calli of *D. major*. (**A**) Fresh weight, (**B**) dry weight, (**C**) growth index (not significant, ns; significant * *p* < 0.05, ** *p* < 0.01, *** *p* < 0.001).

**Figure 2:**
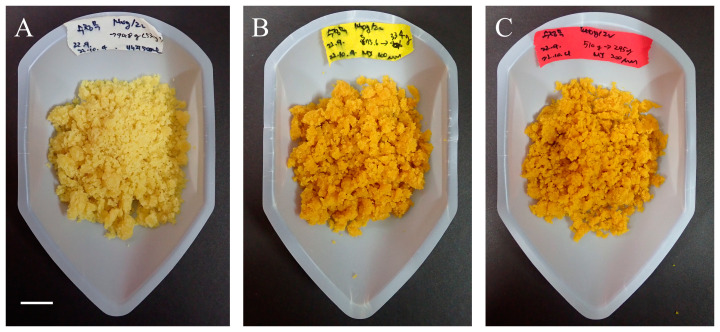
Effect of methyl jasmonate (MeJA) concentration on *D. major* callus browning for 3 weeks. (**A**) Unelicited (control), (**B**) 100 µM MeJA, (**C**) 200 µM MeJA (Scale bar = 1 cm).

Fresh weight was the highest in the control group at 864.0 g, which was 1.5 (100 μM) and 1.7 (200 μM) times higher than that in the MeJA-treated calli (Figure 1A). There was no significant difference between the samples treated with two different MeJA concentrations. The dry weight was 1.3-times higher in the untreated group than in the 200 μM-treated group (Figure 1B). However, as the 100 μM treated group did not show a significant difference from the untreated group, it was considered more advantageous for producing bioactive compounds. Additionally, the color darkened depending on the MeJA concentration (Figure 2). MeJA increases the activity of phenylalanine ammonia lyase (PAL), cinnamate 4-hydroxylase (C4H), chalcone isomerase, stilbene synthase, and anthocyanin synthase, which produce secondary metabolites or inhibit reactive oxygen species (ROS) [18]. Browning is mainly associated with the accumulation of phenolics; phenols upon oxidation form compounds called quinones, which polymerize to give a characteristic brown color and inhibit plant cell growth [19]. Notably, MeJA application to the adventitious roots and calluses of various plants, such as *Ajuga bracteosa* [20], *Thevetia peruviana* [21], and *Mentha × piperita* [22], has been reported to reduce biomass and/or cause browning.

### 2.2. Rubiadin Production through Callus Suspension Cultures

Rubiadin, an anthraquinone found in numerous Rubiaceae family members, has excellent pharmacological properties, including anticancer, anti-inflammatory, antibacterial, antioxidant, and neuroprotective activities [23]. In particular, the chemical properties of rubiadin, a free aglycone with high liposolubility, increase absorption rates in the intestine and induce stronger bioavailability than that obtained with other anthraquinone glycosides [24]. Therefore, we primarily investigated the rubiadin contents in *D. major* leaves and calli.

As shown in Table 1, rubiadin was detected only in the calli of *D. major*. In response to MeJA elicitation, higher levels of rubiadin were observed with increasing MeJA concentration, showing 12.0- and 16.2-times higher levels in the 100 μM and 200 μM MeJA-treated calli than in the control calli. These high–performance liquid chromatograph (HPLC) results were in accordance with previous studies revealing the accumulation of anthraquinones in plant cell cultures with enhanced MeJA elicitation. For instance, Komaraiah et al. determined that 100 μM MeJA maximized the yield of anthraquinones in *Morinda citrifolia* calli, showing four times higher yields than in the control [14]. Perassolo et al. revealed that MeJA treatment induced higher levels of total anthroquinone in *Rubia tinctorum* hairy roots, with an increase of 130% [25]. Zhang et al. reported that 100 μM MeJA treatment in *R. yunnanensis* hairy roots increased the expression of 15 putative genes involved in anthraquinone synthesis, and some of these genes were positively correlated with the contents of rubiquinone and anthraquinone glycoside [26]. In agreement with previously reported results, our data support the idea that MeJA acts as a powerful signal inducer for anthraquinone production in *D. major* calli.

### 2.3. Principal Component Analysis and Metabolite–Metabolite Correlations

Through in vitro callus cultures of *D. major* treated with MeJA, we successfully produced rubiadin, which is a valuable metabolite. In addition to the present findings, MeJA elicitation has also been employed to generate phenolic compounds in various plant cell cultures such as *Thevetia peruviana* [21], *Sageretia thea* [27], and *Cnidium officinale* [28]. Phenolic compounds have attracted considerable attention in culture systems in vitro because they can manipulate biosynthetic routes and accumulate the desired metabolites [29]. They exhibit excellent antioxidant and other pharmacological activities including anti-inflammatory, antibacterial, antiviral, and antifungal properties [30]. However, the biosynthetic pathways of these specialized metabolites are quite complex and are comprehensively correlated with primary metabolites. Therefore, the fundamental quantitative differences in both primary metabolites (amino acids, organic acids, sugars, and sugar alcohols) (Table S1) and specialized metabolites (rubiadin and phenolic compounds) (Table S2) were analyzed to elucidate the overall metabolome patterns in *D. major* leaves and calli.

**Figure 3:**
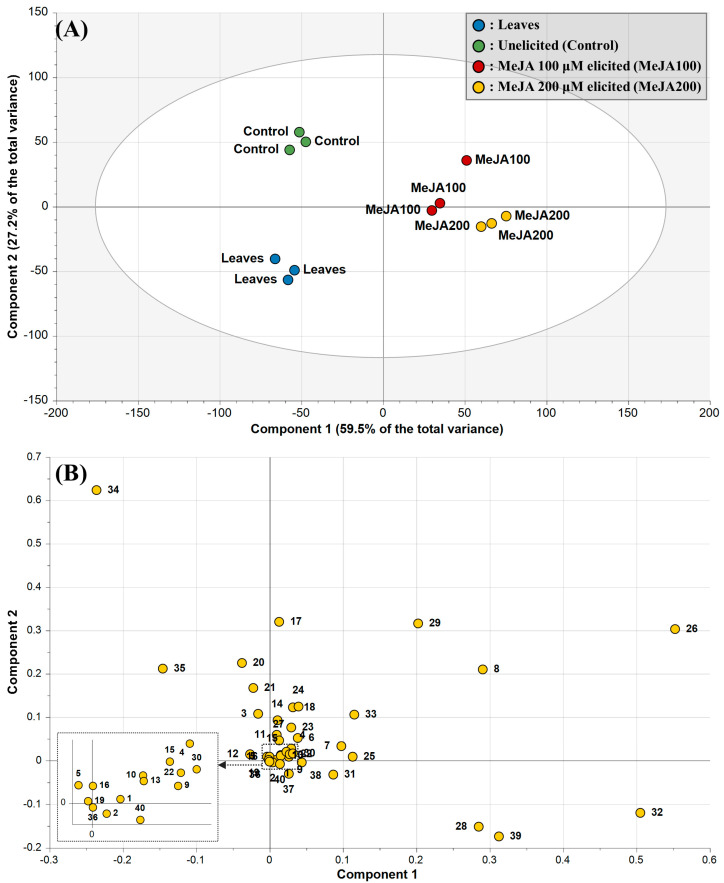
The score (**A**) and loading (**B**) plots of principal components 1 and 2 in the principal component analysis results obtained from *D. major* leaves and calli treated with different methyl jasmonate (MeJA) concentrations. Plot annotations: 1, Lactic acid; 2, Glycolic acid; 3, Alanine; 4, Valine; 5, Urea; 6, Ethanolamine; 7, Glycerol; 8, Phosphoric acid; 9, Leucine; 10, Isoleucine; 11, Glycine; 12, Succinic acid; 13, Fumaric acid; 14, Serine; 15, Threonine; 16, β-Alanine; 17, Malic acid, 18, Aspartic acid; 19, Methionine; 20, Pyroglutamic acid; 21, GABA 22, Threonic acid; 23, Glutamic acid; 24, Phenylalanine; 25, Xylose; 26, Asparagine; 27, Citric acid; 28, Quinic acid; 29, Fructose; 30, Mannose; 31, Galactose; 32, Glucose; 33, Inositol; 34, Sucrose; 35, Raffinnose; 36, Caffeic acid; 37, Epicatechin; 38, Quercetin; 39, Kaempferol; 40, Rubiadin.

Among the various data processing strategies used in metabolomics, principal component analysis (PCA) is a well-known unsupervised dimensionality reduction method and the most widely used multivariate analysis method for extracting interpretative information from a total dataset [31,32]. Thus, the quantification data of 40 primary and specialized metabolites were subjected to PCA for holistic large-scale data analysis (Figure 3A). In the score plot, the two highest-ranking principal components (PCs) accounted for 86.7% of the total variance in the dataset. PC1 accounted for 59.5% of the total variance and separated all elicited *D. major* calli from unelicited *D. major* calli and *D. major* leaves. With PC2, which accounted for 27.2% of the total variance, *D. major* leaves were discriminated from unelicited *D. major* calli. The PCA score plot in the present study provided an overview of the sample clustering patterns but could not clearly distinguish 100 μM and 200 μM MeJA-treated calli. To further investigate the metabolites closely related to the PCs, the corresponding loadings in PC1 and PC2 were compared (Figure 3B). The preferential distribution of metabolites (asparagine, glucose, kaempferol, quinic acid, phosphoric acid, and fructose) in the first and fourth quadrants of the loading plot primarily accounted for differences in the elicited *D. major* calli. Moreover, the distribution in the second quadrant of the loading plot (sucrose and raffinose) represented variation in the unelicited *D. major* calli. In the case of *D. major* leaves, none of the other metabolites appeared to cause significant variation in the third quadrant of the loading plot. This result indicates that most of the primary and secondary metabolites were present at lower levels in *D. major* leaves than in *D. major* calli. This result supports the results of previous studies showing that in vitro calluses accumulate more sugars and amino acids owing to the easy availability of these compounds during their proliferation stage, allowing increased production of secondary metabolites [33,34].

**Figure 4:**
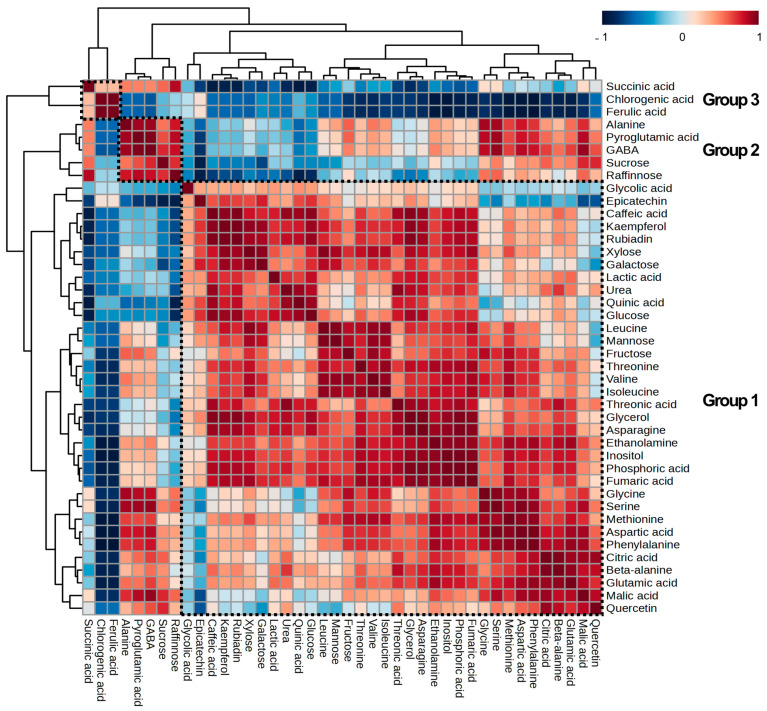
Metabolite to metabolite correlation matrix and hierarchical cluster analysis (HCA) obtained from *D. major* leaves and calli treated with different methyl jasmonate (MeJA) concentrations. Each square indicates the Pearson’s correlation coefficient of a pair of compounds, with the value of this coefficient represented by blue or red colors. A higher intensity of red indicates a positive correlation between metabolites, whereas a higher intensity of blue indicates a negative correlation between metabolites. Each group is marked by a dotted box.

Together with PCA, metabolite–metabolite correlation analysis plays a primary role in indicating the metabolite differences between samples and interpreting metabolite data related to metabolic pathways [35]. To decipher the relationships among 40 identified metabolites in *D. major* leaves and calli, we performed a hierarchical cluster analysis (HCA) using Pearson’s correlation coefficients (Figure 4). Metabolites located close to each other exhibited higher correlations and were represented by red color intensity. In the first group, most metabolites belonging to closely related biochemical pathways, including branched-chain amino acids (valine, leucine, and isoleucine), TCA cycle intermediates (citric acid, malic acid, and fumaric acid), monosaccharides (glucose, galactose, and xylose), and phenolic compounds (kaempferol, epicatechin, and caffeic acid), showed strong correlations. This correlation clustering showed the same pattern as that observed in the PCA loading plots, indicating that all the compounds were positive in PC1. In contrast, pyroglutamic acid, GABA, and alanine were positively correlated with di- and tri-saccharides (sucrose and raffinose) and clustered together in the second group rather than in the first, which included most amino acids. These results were consistent with the PCA results, indicating that these metabolites were located in the second quadrant of the PCA loading plot. Interestingly, both pyroglutamic acid and GABA are glutamic acid-derived amino acids that are used to increase plant resistance to abiotic stress [36,37]. In the third group, succinic acid, chlorogenic acid, and ferulic acid were clustered together. Overall, the observed metabolite–metabolite correlations helped elucidate the PCA results and reflected significant metabolite differences in *D. major* leaves and calli with respect to closely related biological pathways.

### 2.4. Major Metabolite Differences in Response to MeJA Treatment and Concentrations

Although the initial PCA and HCA results provided gross separation among the metabolite data obtained from *D. major* leaves and calli, they had limitations in exploring among-group or within-group variabilities [38]. A previous study reported that partial least squares-discriminant analysis (PLS-DA) is more suitable than PCA for measuring significantly different metabolites when analyzing more than two sample groups [39]. In order to clearly examine the role of MeJA elicitation, we adopted PLS-DA, which allows separation of classes based on their variables, which were unelicited callus, 100 μM MeJA-treated calli, and 200 μM MeJA-treated calli in the present case.

**Figure 5:**
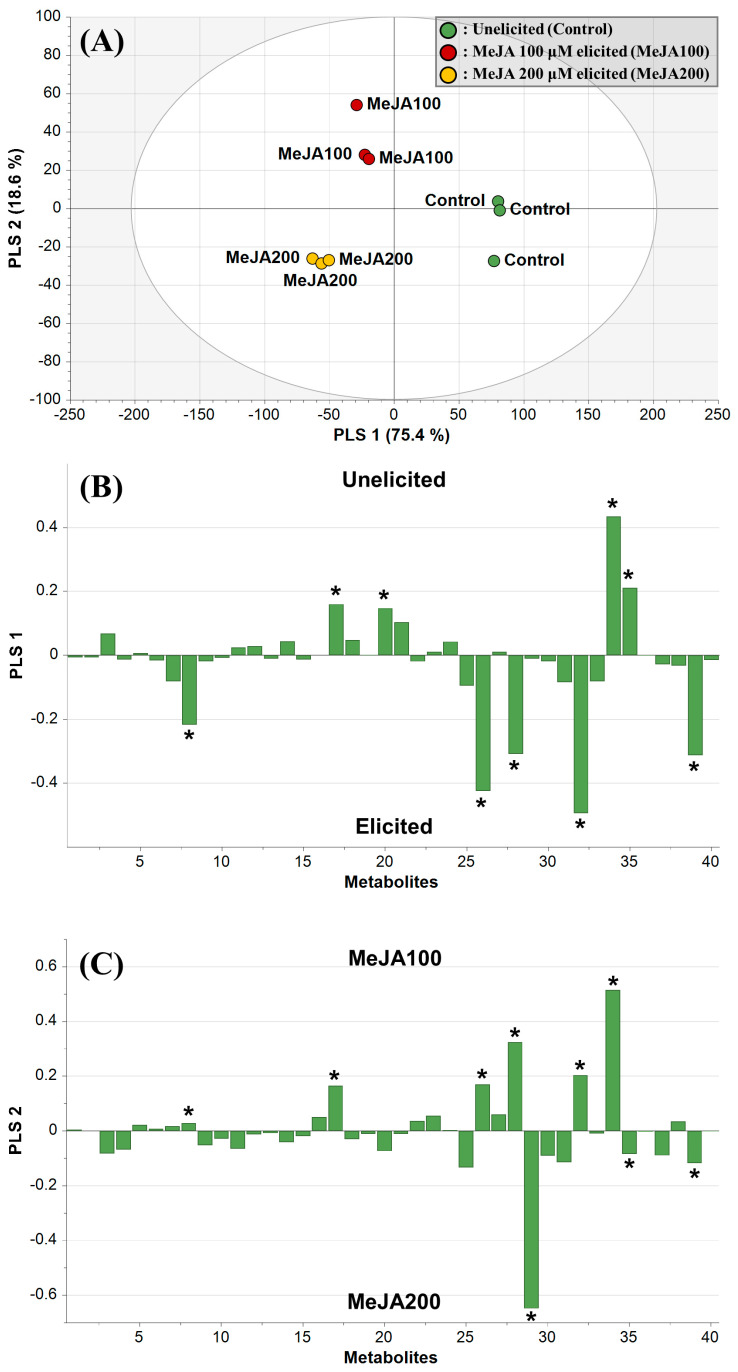
Score plots (**A**) and loading column plots of partial least squares (PLS) 1 (**B**) and 2 (**C**) from the partial least squares discriminant analysis (PLS-DA) of *D. major* leaves and calli treated with different methyl jasmonate (MeJA) concentrations. Asterisk symbols in the loading column plots represent significantly different and influential metabolites with *p* < 0.05 and VIP values ≥ 0.8. Plot annotations: 1, Lactic acid; 2, Glycolic acid; 3, Alanine; 4, Valine; 5, Urea; 6, Ethanolamine; 7, Glycerol; 8, Phosphoric acid; 9, Leucine; 10, Isoleucine; 11, Glycine; 12, Succinic acid; 13, Fumaric acid; 14, Serine; 15, Threonine; 16, β-Alanine; 17, Malic acid, 18, Aspartic acid; 19, Methionine; 20, Pyroglutamic acid; 21, GABA; 22, Threonic acid; 23, Glutamic acid; 24, Phenylalanine; 25, Xylose; 26, Asparagine; 27, Citric acid; 28, Quinic acid; 29, Fructose; 30, Mannose; 31, Galactose; 32, Glucose; 33, Inositol; 34, Sucrose; 35, Raffinnose; 36, Caffeic acid; 37, Epicatechin; 38, Quercetin; 39, Kaempferol; 40, Rubiadin.

The PLS-DA score plot showed clear separation among the samples and explained 94.1% of the total variance, with 89.1% prediction goodness (Figure 5A). These results indicated that the PLS-DA model was reliable. The metabolite profiles of the unelicited and elicited calli were separated using PLS1, accounting for 75.4% of the total variance. In addition, 100 μM and 200 μM MeJA-treated calli were separated by PLS2, accounting for 18.6% of the total variance. In the loading column plot for PLS1, strong metabolites on the positive side with variable importance in the projection (VIP) value ≥ 0.8 included malic acid, pyroglutamic acid, sucrose, and raffinose, indicating higher levels in unelicited calli (Figure 5B and Figure S1A). In contrast, metabolites marked on the negative side of PLS1 with VIP values ≥ 0.8 included phosphoric acid, asparagine, quinic acid, glucose, and kaempferol, which were significant metabolites in elicited calli (both 100 μM and 200 μM MeJA-treated calli) (Figure 5B and Figure S1A). These properties caused by MeJA treatment might be attributed to a shift in the primary metabolism of plant cells toward specialized metabolism, followed by the upregulation of pathogenesis-related genes as well as higher endogenous sugars, and finally, a decrease in cell growth, producing phenolic compounds as defensive compounds (Figure 5B and Figure S2) [40]. As part of specialized metabolism, quinic acid, a phenolic acid derived from the phenylpropanoid pathway, was enhanced by the expression of PAL, C4H, and 4-coumarate CoA ligase in response to MeJA elicitation, as revealed in *Gardenia jasminoides* cells [41]. Moreover, endogenous phenolic acids triggered the synthesis of flavonoids by inducing oxidative stress in *Coriandrum sativum* calli [42]. In sugar metabolism, sucrose and raffinose are the commonly used di- and tri-saccharide carbohydrate sources, respectively, required for flavonoid production [43,44]. Consistent with previous studies, subsequent glycolysis and gluconeogenesis after the exogenous application of phytohormones also increased glucose content to facilitate defense mechanisms [45,46]. Higher levels of asparagine and lower levels of pyroglutamic acid and malic acid were observed after MeJA treatment. Similarly, a recent investigation involving the metabolite profiling of elicited *Salvia miltiorrhiza* showed upregulated asparagine and glutamic acid, implying that the conversion of glutamic acid to pyroglutamic acid was stopped [46]. When elicited with MeJA, a lower concentration of malic acid might be linked with the utilization of precursors in other related pathways; however, this cannot be clearly explained by their ecological functions or through common catabolic phenomena, such as protein degradation [47]. Consistent with these previous studies, our results indicated that MeJA elicitation reorganized various primary metabolites (including central carbons, amino acids, and sugars) as well as specialized metabolites in *D. major* calli, and that these metabolic strategies were significantly linked to defensive mechanisms against elicitation.

Considering the effects of MeJA concentration, a loading column plot for PLS2 marked on positive side with VIP values ≥ 0.8 indicated the main metabolites that were significantly higher in 100 μM MeJA-treated calli, including sucrose, quinic acid, glucose, asparagine, malic acid, and phosphoric acid (Figure 5C and Figure S1B). Further, the metabolites presenting strong negative values of PLS2 with VIP values ≥ 0.8 included fructose, raffinose, and kaempferol, explaining their significantly higher values in 200 μM MeJA-treated calli (Figure 5C and Figure S1B). Consistent with the above results regarding metabolic changes induced by MeJA, kaempferol content increased and that of sucrose and malic acid decreased in response to elevated MeJA treatment concentrations (Figure 5C and Figure S2). Related to the morphogenic characteristics of *D. major* calli under elicitation with 200µM MeJA, our results showed that excessive MeJA treatment reduced the growth index and that the calli underwent oxidative stress, which overwhelmed their own antioxidant capabilities (Figure 1). Further, the metabolite differences presented in PLS2 were that *D. major* calli treated with 200µM MeJA contained higher levels of raffinose and fructose but lower levels of glucose, quinic acid, asparagine, and phosphoric acid, compared with those in calli treated with 100 µM MeJA (Figure 5C and Figure S2). Although MeJA is widely utilized as a signaling molecule for regulating plant growth and the production of specialized metabolites, over-exploitation of MeJA often leads to cell growth inhibition, which might be caused by inhibitory photosynthesis [48]. These results are further supported by those of ElSayed et al., who reported that raffinose levels in chloroplasts were significantly higher upon exposure to excessive abiotic stress [49]. Consistent with our results, another report mentioned that although both fructose and glucose were decomposition products of sucrose, glucose was utilized for monosaccharide metabolism in *Salvia miltiorrhiza* cells under prolonged exposure to the elicitor [50]. Another study also reported that asparagine metabolism was upregulated with 100 µM MeJA to activate the aminoacyl-tRNA biosynthesis pathway, which is a critical pathway in the plant stress response and is responsible for the attachment of amino acids such as asparagine and 4-aminobutyrate [51]. However, this pathway was dramatically decreased upon adding 200 µM MeJA as asparagine was altered to stimulate specialized metabolite production [51]. As 200 µM MeJA increased the accumulation of asparagine, which acts as an enzyme stimulator, quinic acid entered the phenylpropanoid pathway and was altered to other specialized metabolites [52,53]. Thus, MeJA dose-response experiments were important for discovering an efficient alternative method for producing specialized metabolites with improved cell growth rates.

## 3. Materials and Methods

### 3.1. Callus Production and Elicitation

*D. major* calli were maintained in suspension cultures in Murashige and Skoog (MS) [54] liquid medium supplemented with 16.11 μM α-naphthaleneacetic Acid (NAA), 0.46 μM kinetin, and 3% sucrose. A 5 L bioreactor containing 2.5 L of medium was cultured with 70.0 g fresh weight (FW)/L calli, and sterilized air was continuously supplied at 0.1 vvm (aeration volume/medium volume/minute). Methyl jasmonate (MeJA, Sigma Chemical Co., MO, USA) stock solutions (1000 μM) were prepared in absolute ethanol and filter-sterilized. MeJA was added to *D. major* calli after 2 weeks of culture at 100 μM or 200 μM. The two most commonly used concentrations of MeJA (100 μM and 200 μM) were chosen on the basis of previous studies (Table S3). The calli were then harvested after 1 week of elicitor treatment. All cultures were maintained at 25 ± 1 °C in the dark. The callus biomass was measured after 3 weeks of culture, and the fresh weight (FW) was determined after washing the calli with distilled water and removing excess surface water. Dry weight (DW) was recorded after drying the callus in an oven at 40 °C for 48 h. The growth index (GI) was calculated as follows:

GI = [Final DW (g) − Initial DW (g)]/Initial DW (g)

### 3.2. Preparation of Plant Materials

*D. major* leaves were collected from Seogwipo-si, Jeju, Republic of Korea, on 30 September 2022. The harvested leaves and calli were dried using an oven at 40 °C and then ground to fine powder in a mechanical grinder. Prior to compound extraction, each sample was passed through a 200 μm sieve. For each analysis, three biological replicates were analyzed under identical conditions.

### 3.3. Extraction and Analysis of Rubiadin

Rubiadin analysis was conducted as previously described with some modifications [55,56]. Dried and ground *D. major* leaves and calli (approximately 10 g) were extracted with 200 mL of 70% ethanol (*v*/*v*) for 24 h in a rotary shaker. After filtering through a filter paper (No. 2; Advantec, Tokyo, Japan), the extracts were evaporated and freeze-dried. Powdered sample extracts (10 mg) were dissolved in 1 mL of 50% methanol (*v*/*v*) and sonicated for 10 min. The final extracts were then filtered through a 0.50 µm PTFE syringe filter (13JP010AN; Advantec) for HPLC analysis. Chromatographic separation was performed on a Waters Alliance e2695 HPLC system (Waters Corp., Milford, MA USA) equipped with a photodiode array (PDA) detector, and a Sunfire C18 column (250 × 4.6 mm, 5 μm particle size; Waters Corp., Milford, MA, USA). The injection volume was 10 µL, and the column temperature was maintained at 40 °C. The flow rate was set at 1 mL/min. The solvent gradient was formed using 0.1% of phosphoric acid in water (*v*/*v*) as solvent A and acetonitrile as solvent B, and was consecutively programmed as follows: 0 min–20% B; 10 min–20% B; 30 min–100% B; 40 min–100% B; 41 min–20% B; 50 min–20% B. HPLC chromatograms were monitored in the wavelength range of 200–400 nm. For quantitative analysis, peaks were obtained at 250 nm and identified by comparing the retention times and PDA spectra with those of an authentic standard. An external calibration curve was constructed by loading rubiadin in the range of 2.5–50 μg/mL. Chromatographic data were obtained using Empower 3 software (Waters Corp.).

### 3.4. Extraction and Analysis of Phenolic Compounds

The phenolic compounds were extracted and analyzed as described in our previous study, with slight modifications [57,58]. For extracting phenolic compounds, 70 mg of powdered samples was weighed and placed in 2 mL tubes. To this, 1 mL of methanol and 200 μL of 0.1 M hydrochloric acid (HCl) were added followed by sonication for 1 h. The samples were centrifuged at 4 °C and 15,000 rpm for 10 min, and then 850 μL of the supernatants was transferred to fresh tubes. The remaining pellet was re-extracted with methanol (1 mL) and sonicated for 1 h. After centrifugation at 4 °C and 15,000 rpm for 10 min, 1 mL of the supernatants was concentrated together with 850 μL of previously collected supernatants using a centrifugal evaporator (CVE-3000; EYELA, Tokyo, Japan). The resulting extracts were dissolved in 1 mL of methanol and then filtered through a 0.50 µm PTFE syringe filter (Adventac). For the analysis, 10 μL of filtrate was injected into a Waters Alliance e2695 HPLC system (Waters Corp.) coupled with a PDA detector. Separations were performed on a Zorbax SB-C18 column (250 × 4.6 mm, 5 μm particle size; Agilent, Palo Alto, CA, USA) at 40 °C with a flow rate of 1 mL/min. Mobile phase solvents A and B were 0.1% acetic acid in water (*v*/*v*) and acetonitrile, respectively. The solvent gradient conditions were programmed as follows: 0 min–8% B; 2 min–10% B; 50 min–28% B; 55 min–92% B; 60 min–92% B; 70 min–8% B. Chromatograms were observed in the range of 200–400 nm, and the detection wavelength was set at 280 nm. Each compound was identified by matching the retention times and spectral characteristics to those of authentic standards. Each analysis was externally calibrated by loading standard solutions with concentrations ranging from 2.5 to 80 μg/mL. Data were acquired using Empower 3 software (Waters Corp.).

### 3.5. Extraction and Analysis of Low-Molecular Hydrophilic Compounds

Low-molecular-weight hydrophilic compounds (including amino acids, organic acids, sugars, and sugar alcohols) were extracted and analyzed using our previously reported methods [57,58]. For extraction, 1 mL of a methanol:chloroform:water (2.5:1:1, *v*/*v*/*v*) solution was added to 15 mg of each sample. Subsequently, 60 μL of adonitol (200 μg/mL in methanol) was added as an internal standard (IS). The samples were then vortexed and incubated in a thermo shaker at 37 °C and 1200 rpm for 30 min. After centrifugation at 4 °C and 13,000 rpm for 3 min, 800 μL of supernatant was transferred into fresh tubes, and 400 μL of water was added. The mixed samples were centrifuged under the conditions described above. Next, 900 μL of the upper methanol/water phase was transferred into fresh tubes and then dried in a centrifugal concentrator (CVE-3000; EYELA) for 3 h. After lyophilization for 16 h, methoxime derivatization was performed by adding 80 μL of methoxyamine hydrochlorides (20 mg/mL in pyridine) and incubating in a thermo shaker at 30 °C and 1200 rpm for 90 min. Trimethylsilyl esterification was subsequently performed by introducing 80 μL of *N*-methyl-*N*-(trimethylsilyl) trifluoroacetamide and incubating in a thermo shaker at 37 °C and 1200 rpm for 30 min. The derivatized samples were analyzed using an Agilent 7890A gas chromatograph (GC) (Agilent, Palo Alto, CA, USA) equipped with an Agilent 5975C mass spectrometer (MS) on a CP-Sil 8CB low-bleed/MS fused-silica capillary column (30 m × 0.25 mm i.d. × 0.25 μm film thickness; Agilent). For each analysis, 1 μL of sample was injected with a split ratio of 25:1. The inlet temperature was set at 230 °C. Helium was used as the carrier gas, flowing at a constant rate of 1.00 mL/min. The column temperature was programmed as follows: the initial temperature was set at 80 °C for 2 min, followed by ramping to 320 °C at 15 °C/min, and holding at 320 °C for 10 min. The MS quadrupole and ion source temperatures were set as 150 °C and 230 °C, respectively. Mass spectra were scanned in the range of 85–600 *m/z*. The ionization voltage was set as 70 eV. Peaks were identified by comparing the mass spectral data of each sample with those of the NIST and in-house libraries. For quantification, the ratios of the analyte to the IS peak area were calculated for selected ions.

### 3.6. Statistical Analysis

All analyses were performed in triplicate. The experimental data for rubiadin were analyzed using analysis of variance (ANOVA), and significant differences among means were identified using Tukey’s multiple-range test (GraphPad Prism 8; San Diego, CA, USA). Using soft independent modeling of class analogy (SIMCA) software (version 17.0, Umetrics, Umeå, Sweden), all data were normalized using Pareto scaling; PCA and PLS-DA were subsequently performed to reveal relationships among or within group variances. HCA and Pearson’s correlation analyses were performed using MetaboAnalyst 5.0 (https://www.metaboanalyst.ca (accessed on 4 December 2023)) to identify metabolite–metabolite correlations. For pathway analysis, the normalized metabolic abundance data were visualized on a metabolic pathway through Pathvisio Software (version 3.3.0, http://www.pathvisio.org, Maastricht University, Maastricht, The Netherlands (accessed on 4 December 2023)).

## 4. Conclusions

Overall, this study described the metabolic differences between *D. major* leaves and callus cultures treated with different concentrations of MeJA. First, we compared the growth characteristics of *D. major* calli to determine suitable MeJA concentrations for high production rates. We found that there was no significant difference in the biomass reduction of *D. major* calli upon treatment with 100 µM MeJA, and that browning occurred in a MeJA concentration-dependent manner. Using the callus culture technique with MeJA elicitation, we produced rubiadin, an anthraquinone with various biological activities. Rubiadin was not observed in *D. major* leaves; however, the rubiadin content in *D. major* calli increased with increasing levels of MeJA. Further, multivariate analysis to distinguish significant differences in the levels of primary and specialized metabolites in *D. Major* leaves and calli treated with different elicitors revealed that the in vitro calli had higher amino acid, sugar, and phenolic compound levels than those in *D. major* leaves, explained by the availability of these primary metabolites in their proliferation stage; these then stimulated the production of functional specialized metabolites. Further, our results indicated that MeJA elicitation contributed to activate the production of kampferol and quinic acid as defensive mechanisms, followed by degradation of sucrose and raffinose to glucose for exploiting them as energy sources. Moreover, elicitation decreased the conversion to pyroglutamic acid and increased asparagine metabolism. Concerning the MeJA treatment concentration, 200 µM MeJA-treated calli also featured high amounts of asparagine, which was ascribed to aminoacyl-tRNA biosynthesis pathway activated by the attachment of asparagine to respective transfer RNAs. Moreover, lower levels of glucose and sucrose and a higher level of fructose were observed in the 200 µM MeJA-treated calli, because glucose is more utilized than fructose as a decomposition product of sucrose in monosaccharide metabolism with excessive stress. Overall, our results suggest that 100 µM MeJA treatment of *D. Major* calli can be the best strategy for increasing valuable specialized metabolites with maintained production rates. The obtained insights will facilitate optimization of plant cell culture techniques for improved biosynthesis of anthraquinones and flavonoids.

## Figures and Tables

**Table 1:** One-way analysis of variance (ANOVA) results for the content of rubiadin in *D. major* leaves and calli treated with different methyl jasmonate (MeJA) concentrations.

(mg/g Dry Weight)	Leaves	Unelicited	Elicited	
		Control	MeJA 100	MeJA 200
Rubiadin	* N.D. ^a^	0.121 ± 0.007 ^b^	1.458 ± 0.008 ^c^	1.961 ± 0.143 ^d^

* N.D. Not detected; ^a,b,c,d^ Different superscript letters indicate statistical differences with *p* < 0.05 (Tukey’s multiple-range test).

## Data Availability

The data presented in this study are available upon request from the corresponding author.

## Supplementary Materials

The following supporting information can be downloaded at: https://www.mdpi.com/article/10.3390/plants13020167/s1, Figure S1: The influence of variables in projection plots displaying contributed metabolites to construct partial least squares (PLS) 1 (A) and 2 (B) from partial least squares discriminant analysis (PLS-DA) results obtained from leaves and calli treated with different methyl jasmonate (MeJA) concentrations; Figure S2: Metabolic pathways of *D. major* calli treated with various methyl jasmonate (MeJA) concentrations. Metabolite differences were visualized as the scaling factor ranges from −1.8 to 1.8. A scaling value above zero represents higher than average levels in each value of the control, MeJA 100 μM-treated calli, and MeJA 200 μM-treated calli, and is colored by the red color intensity. A scaling value below zero represents lower than the average levels and is indicated by green color intensity. Gray color represents not detected metabolites; Table S1: Composition and content (ratio/g) of low-molecular hydrophilic compounds in *D. major* leaves and calli treated with different methyl jasmonate (MeJA) concentrations; Table S2: Composition and content (μg/g) of phenolic compounds in *D. major* leaves and calli treated with different methyl jasmonate (MeJA) concentrations; Table S3: Suitable concentrations of methyl jasmonate (MeJA) in cell cultures of various plants. References [59,60,61,62,63,64,65,66,67,68,69,70] are cited in the supplementary materials.
