# Supplementary material for: Metabolomics Reveals Rubiadin Accumulation and the Effects of Methyl Jasmonate Elicitation in Damnacanthus major Calli
Source: Plants (Basel). 2024 Jan 8;13(2):167. doi: 10.3390/plants13020167 (PMC10820265; doi:10.3390/plants13020167)
Supplement: Supplementary file 1 [file plants-13-00167-s001.zip › plants-2786852-supplementary.pdf]

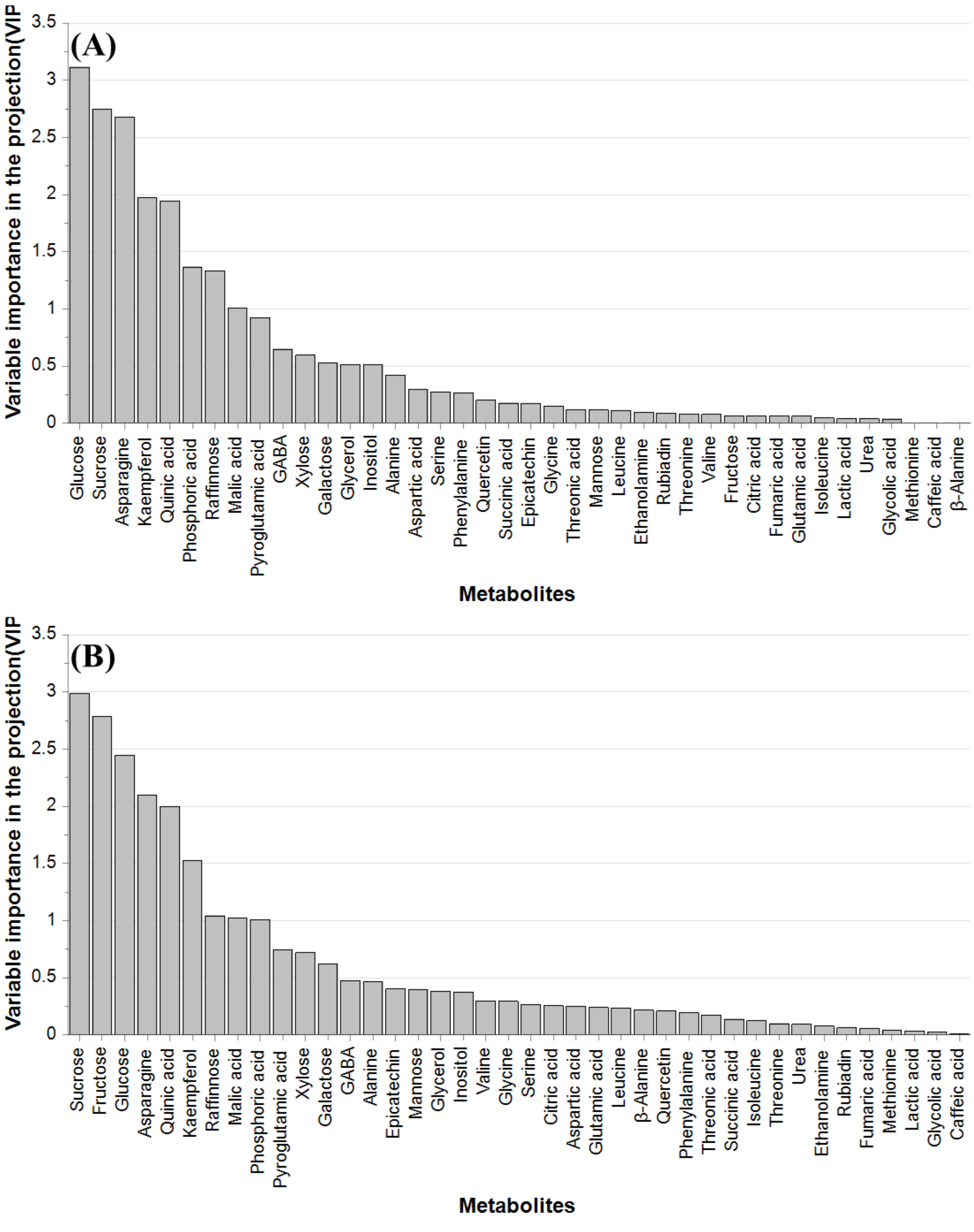

Figure S1. The influence of variables in projection plots displaying contributed metabolites to construct partial least squares (PLS) 1 (A) and 2 (B) from partial least squares discriminant analysis (PLS-DA) results obtained from leaves and calli treated with different methyl jasmonate (MeJA) concentrations

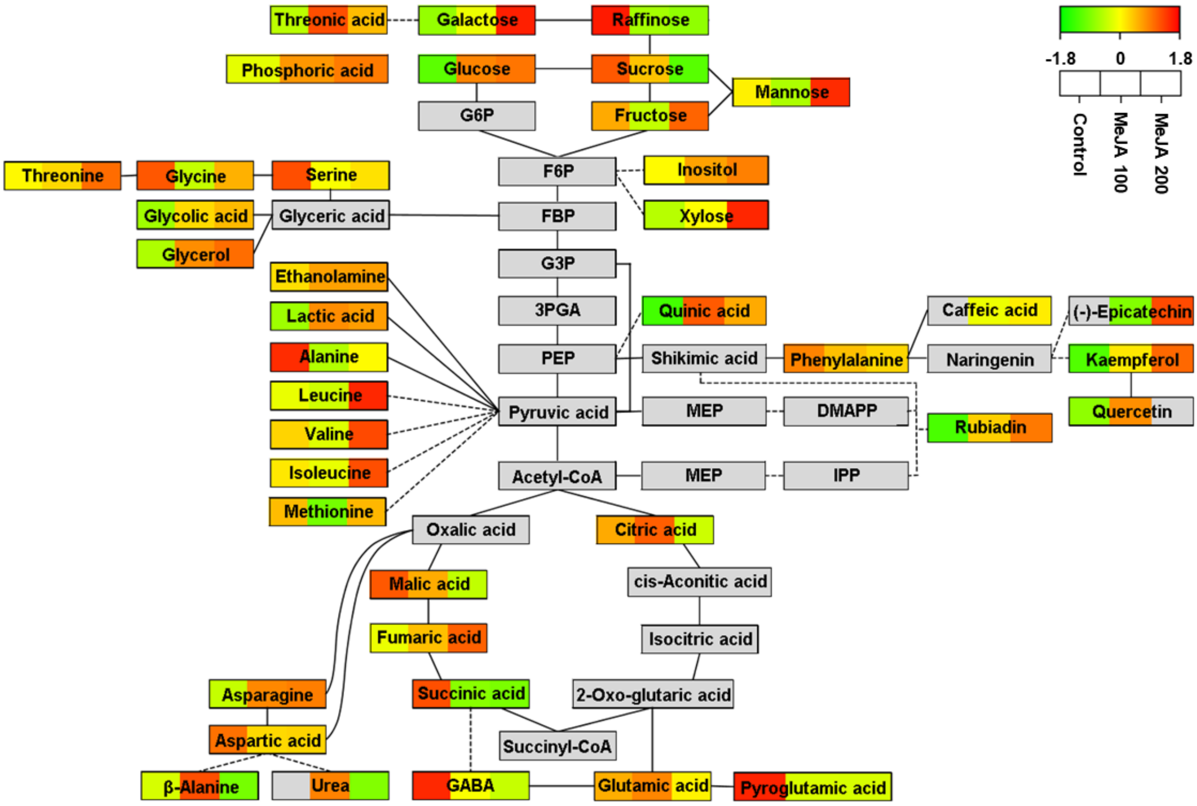

Figure S2. Metabolic pathways of *D. major* calli treated with various methyl jasmonate (MeJA) concentrations. Metabolite differences are visualized as the scaling factor ranges from -1.8 to 1.8. A scaling value above zero represents higher than average levels in each value of the control, MeJA 100 μM-treated calli, and MeJA 200 μM-treated calli, and is colored by the red color intensity. A scaling value below zero represents lower than the average levels and is indicated by green color intensity. Gray color represents not detected metabolites.

Table S1. Composition and content (ratio/g) of low-molecular hydrophilic compounds in *D. major* leaves and calli treated with different methyl jasmonate (MeJA) concentrations.

Compounds	Leaves	Unelicited	Elicited	
		Control	MeJA 100	MeJA 200
		Amino acids		
Alanine	5.706 ± 1.431	70.169 ± 15.24	16.741 ± 2.033	31.648 ± 4.085
Asparagine	1.896 ± 0.449	1097.804 ± 259.299	2581.31 ± 550.039	2671.850 ± 243.076
Aspartic acid	2.256 ± 0.284	75.391 ± 3.940	54.379 ± 5.150	55.540 ± 6.360
GABA	40.105 ± 2.525	160.538 ± 6.091	88.867 ± 7.346	71.662 ± 7.447
Glutamic acid	1.257 ± 0.325	27.913 ± 7.048	31.552 ± 6.172	21.619 ± 3.276
Glycine	0.765 ± 0.155	21.365 ± 0.408	8.196 ± 1.158	16.122 ± 2.249
Isoleucine	0.774 ± 0.379	3.181 ± 0.187	2.653 ± 0.619	5.029 ± 1.281
Leucine	0.471 ± 0.265	4.086 ± 0.535	3.291 ± 0.883	10.781 ± 1.418
Methionine	*N.D.	1.292 ± 0.049	1.005 ± 0.156	1.300 ± 0.219
Phenylalanine	1.649 ± 0.077	76.978 ± 1.965	64.196 ± 6.594	59.271 ± 4.918
Pyroglutamic acid	2.878 ± 1.542	250.197 ± 40.371	75.168 ± 5.143	73.805 ± 2.462
Serine	0.918 ± 0.382	41.91 ± 1.444	22.026 ± 2.505	25.726 ± 4.983
Threonine	0.824 ± 0.268	5.348 ± 1.606	6.119 ± 1.255	8.600 ± 2.598
Valine	1.517 ± 0.550	12.535 ± 1.103	8.966 ± 0.908	19.106 ± 0.984
β-Alanine	N.D.	7.760 ± 0.176	11.071 ± 0.787	6.296 ± 0.323
		Organic acids		
Citric acid	2.565 ± 0.287	12.482 ± 0.184	15.202 ± 0.082	7.684 ± 0.129
Fumaric acid	0.502 ± 0.203	1.868 ± 0.060	2.481 ± 0.295	3.191 ± 0.300
Glycolic acid	1.488 ± 0.814	1.221 ± 0.197	1.632 ± 0.243	1.733 ± 0.306
Lactic acid	0.919 ± 0.231	0.968 ± 0.227	1.550 ± 0.430	1.505 ± 0.211
Malic acid	13.794 ± 1.489	434.075 ± 13.652	333.995 ± 17.559	162.101 ± 8.805
Quinic acid	373.541 ± 44.299	21.653 ± 0.446	998.035 ± 63.116	777.675 ± 29.351
Succinic acid	10.244 ± 1.720	12.424 ± 0.537	6.096 ± 0.703	5.784 ± 0.487
Threonic acid	0.874 ± 0.136	3.512 ± 0.791	9.531 ± 1.348	6.832 ± 0.481
Urea	N.D.	N.D.	3.575 ± 0.245	2.169 ± 0.244
		Sugars and sugar alcohols		
Fructose	552.877 ± 44.456	1606.005 ± 387.777	1031.473 ± 64.484	1886.648 ± 111.188
Galactose	12.775 ± 0.918	11.194 ± 0.633	33.587 ± 9.758	95.657 ± 11.519
Glucose	1428.6 ± 180.606	975.262 ± 99.936	2879.966 ± 439.298	2983.979 ± 150.923
Glycerol	12.294 ± 1.405	36.208 ± 1.315	83.541 ± 6.210	91.424 ± 6.290
Inositol	29.171 ± 0.973	133.579 ± 5.643	174.079 ± 1.547	190.520 ± 2.500
Mannose	3.846 ± 0.110	13.332 ± 1.011	7.463 ± 1.767	25.408 ± 1.905
Raffinnose	136.884 ± 14.705	410.321 ± 16.908	70.797 ± 14.930	49.551 ± 2.261
Sucrose	2436.117 ± 389.194	3945.104 ± 281.663	3232.052 ± 715.845	1707.375 ± 34.724
Xylose	1.509 ± 0.255	29.436 ± 5.061	56.294 ± 5.949	137.218 ± 8.916

		Others		
Ethanolamine	6.378 ± 1.182	22.427 ± 0.897	26.296 ± 5.589	26.354 ± 2.671
Phosphoric acid	5.446 ± 0.501	473.446 ± 19.007	797.682 ± 65.939	870.452 ± 46.314

* N.D. Not detected.

Table S2. Composition and content (μg/g) of phenolic compounds in *D. major* leaves and calli treated with different methyl jasmonate (MeJA) concentrations.

Compounds	Leaves	Unelicited	Elicited	
		Control	MeJA 100	MeJA 200
Caffeic acid	*N.D.	N.D.	12.851 ± 0.354	13.014 ± 1.002
Chlorogenic acid	1699.363 ± 21.422	N.D.	N.D.	N.D.
Epicatechin	33.958 ± 1.118	N.D.	28.871 ± 0.481	51.713 ± 4.012
Ferulic acid	21.077 ± 0.14	N.D.	N.D.	N.D.
Kaempferol	N.D.	66.262 ± 3.75	592.774 ± 6.625	935.772 ± 9.931
Quercetin	N.D.	47.035 ± 0.801	N.D.	N.D.

* N.D. Not detected.

Table S3. Suitable concentrations of methyl jasmonate (MeJA) in cell cultures of various plants.

Plant	Materials	MeJA concentration	Reference
Glycyrrhiza glabra	Callus	100 µM	[59]
Hypericum perforatum	Callus	100 µM	[60]
Malus sieversii f. niedzwetzkyana	Callus	100 µM	[61]
Panax ginseng	*CSC	200 µM	[62]
Silybum marimum	CSC	100 µM	[63]
Morinda citrifolia	CSC	150 µM	[14]
Panax notoginseng	CSC	200 µM	[64]
Eschscholtzia californica	CSC	100 µM	[65]
Mentha × piperita	CSC	100 µM	[22]
Satureja khuzistanica	CSC	100 µM	[66]
Changium smyrnioides	CSC	100 µM	[67]
Persicaria minor	CSC	100 µM	[68]
Gardenia jasminoides	CSC	200 µM	[69]
Orostachys cartilaginous	CSC	100 µM	[70]

* CSC: Cell suspension culture
